# A case report of a patient with bulky uterine cervical neoplasm who achieved complete response with “intentional internal high-dose policy” high-dose-rate interstitial brachytherapy

**DOI:** 10.1097/MD.0000000000020860

**Published:** 2020-07-02

**Authors:** Tairo Kashihara, Kazuma Kobayashi, Kotaro Iijima, Naoya Murakami, Ken Yoshida, Kae Okuma, Satoshi Nakamura, Kana Takahashi, Koji Inaba, Hiroshi Igaki, Yuko Nakayama, Tomoyasu Kato, Takashi Uno, Jun Itami

**Affiliations:** a Department of Radiation Therapy, National Cancer Center Hospital, Tokyo; b Department of Radiology, Osaka Medical College Hospital, Osaka; c Department of Gynecologic Oncology, National Cancer Center Hospital, Tokyo; d Department of Radiology, Chiba University Hospital, Chiba, Japan.

**Keywords:** ablative radiation therapy, brachytherapy, bulky tumor, gynecological neoplasm, uterine cervical neoplasm

## Abstract

**Rationale:**

Gynecological high-dose-rate (HDR) brachytherapy has progressed for years, but it remains difficult for bulky tumors to be controlled locally. Dose limitations to organs at risk (OARs) are invariably obstacles in increasing the prescription dose. Additionally, it is controversial that the excessive hyperdose sleeve, the volume receiving a dose equal to or greater than twice the reference dose, should be eliminated in gynecological HDR brachytherapy. On the other hand, the technique of simultaneous integrated protection was reported for large hepatocellular carcinoma treatment, and similarly, internal high-dose brachytherapy could be used for treating bulky cervical carcinoma.

**Patient Concerns:**

A 54-year-old female had irregular genital bleeding and lost 13 kg in one year.

**Diagnosis:**

She was diagnosed with T3bN1M0 cervical cancer in another hospital. The transverse diameter of the primary tumor was 10.5 cm.

**Interventions:**

The whole pelvis and para-aortic lymph node were irradiated with a total of 50 Gy in 25 fractions, but the size of the tumor showed only a slight decrease to 8.9 cm. After external beam radiotherapy, first-time high-dose-rate interstitial brachytherapy (HDR-ISBT) was administered without “intentional internal high-dose (IIHD) policy,” the technique of high-dose administration to only the inside of the tumor. Considering the rectum dose limitation, in the additional 2 times of brachytherapy, “IIHD policy” HDR-ISBT was applied. In the second and third HDR-ISBT, the percentage of the volume exposed to 200% of the prescribed dose for high-risk clinical target volume increased by 241% and 204% compared with the first HDR-ISBT, while the doses to OARs were not significantly higher than those of the first-time HDR-ISBT.

**Outcomes:**

Complete response was obtained, and no recurrence findings and side effects caused by HDR-ISBT have been detected for 2 years and 9 months.

**Lessons:**

To our knowledge, this is the first report of IIHD HDR-ISBT for bulky cervical cancer. This technique can be the solution for treating bulky cervical cancer.

## 1 Introduction

Gynecological high-dose-rate (HDR) brachytherapy has progressed for years through the introduction of image-guided brachytherapy. Nonetheless, it remains difficult for locally advanced cervical cancer to be controlled locally.^[1]^ Though interstitial brachytherapy (ISBT) is known to bring high local control,^[2]^ Itami et al^[3]^ revealed that local control was significantly worse (25% vs 69%) in case the size of tumor was more than 6 cm in gynecological HDR-ISBT. Furthermore, it has been reported that lower tumor doses lead to inferior local control rate.^[4,5]^ Therefore, to control tumor locally, enhancing the doses to the tumor is desirable. In contrast, dose–volume relationships have been reported on small bowel, rectum, and urinary adverse events.^[6–8]^ Dose limitations to organs at risk (OARs) are invariably obstacles in increasing the prescription dose; therefore, there has been no systematic dose escalation study for cervical cancer.

Meanwhile, in recent years, hypofractionated radiotherapy has become a common treatment method, and the question whether internal high-dose distribution in the tumor is better or not arises. In head and neck brachytherapy, it has been demonstrated that the excessive hyperdose sleeve, the volume receiving a dose equal to or greater than twice the reference dose, should be eliminated in HDR brachytherapy.^[9]^ On the other hand, the policy of simultaneous integrated boost (SIB) with simultaneous integrated protection (SIP) was reported for large hepatocellular carcinoma.^[10]^ This policy means enhancing irradiation dose to the tumor while saving OARs with SIB. However, for gynecological ISBT, no study of internal high-dose distribution has been reported thus far. We assumed that the policy of administering high dose to only the inside of the tumor in saving OARs in HDR-ISBT could be the solution for bulky cervical carcinoma to control the primary tumor safely. We named this technique as “intentional internal high-dose (IIHD) policy” HDR-ISBT in contradistinction to conventional “homogeneous dose distribution (HDD) policy” HDR-ISBT. Herein, we present a case of bulky cervical cancer treated with this “IIHD policy” HDR-ISBT safely and effectively.

## 2 Case presentation

A 54-year-old female was diagnosed with cervical clear cell carcinoma in another hospital. The tumor-node-metastasis stage according to the UICC 8th edition was clinical T3bN1M0, and bilateral parametrial infiltration was detected. The transverse maximum diameter of the primary tumor was 10.5 cm (volume, 532 cm^3^). A total of 50 Gy in 25 fractions for the whole pelvis and para-aortic lymph node was irradiated using the 2 opposed-fields technique external beam radiation therapy, but the size of the primary tumor decreased by only 1.6 cm (the tumor volume, 393 cm^3^). HDR-ISBT was recommended to control the primary tumor, and then, she was referred to our institution. First-time HDR-ISBT was administered without IIHD policy. The procedure of ISBT for patients with cervical cancer has been described by Murakami et al and Kashihara et al.^[11–13]^

The first ISBT was performed with “HDD policy.” The maximum dose to the most exposed 0.1 cc (D0.1 cc) and 2.0 cc (D2.0 cc) for the rectum, sigmoid and bladder (rectum D0.1cc/2.0cc, sigmoid D0.1cc/2.0cc and bladder D0.1cc/2.0cc) and the dose indexes for high-risk clinical target volume (HRCTV) coverage (HRCTV D90/V100) were evaluated in the first ISBT. Rectum D0.1cc/2.0cc was 572 cGy/491 cGy and HRCTV D90 and V100/200/300/400/500 were 630 cGy and 93.8%/13.8%/4.2%/2.1%/1.2%, respectively. In our institution, the criteria of the rectum D2.0cc < 75 Gy have been adopted as a rectum dose limitation in reference to the prospective multicenter EMBRACE (Image guided intensity modulated external beam radiochemotherapy and MRI based adaptive brachytherapy in locally advanced cervical cancer) study.^[14]^ Considering this rectum dose limitation, additional 2 times of brachytherapy can be tolerable after the first time HDR-ISBT. On the other hand, the primary tumor would not be controlled with “HDD policy” HDR-ISBT because the tumor diameter was still greater than 6 cm.^[3]^ Therefore, “IIHD policy” HDR-ISBT was adopted to control the bulky primary tumor safely.

**Figure 1:**
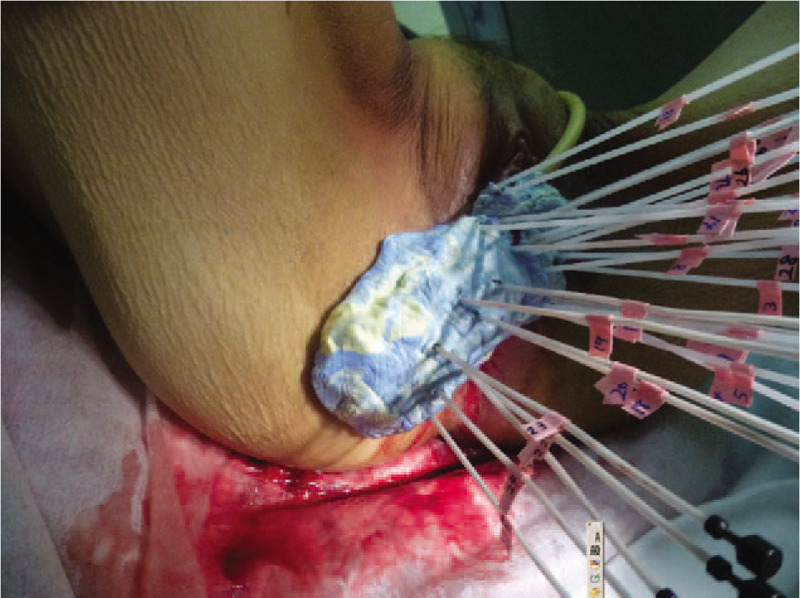
Needle fixation. The needles were fixed with silicon putty for laboratory purposes.

**Figure 2:**
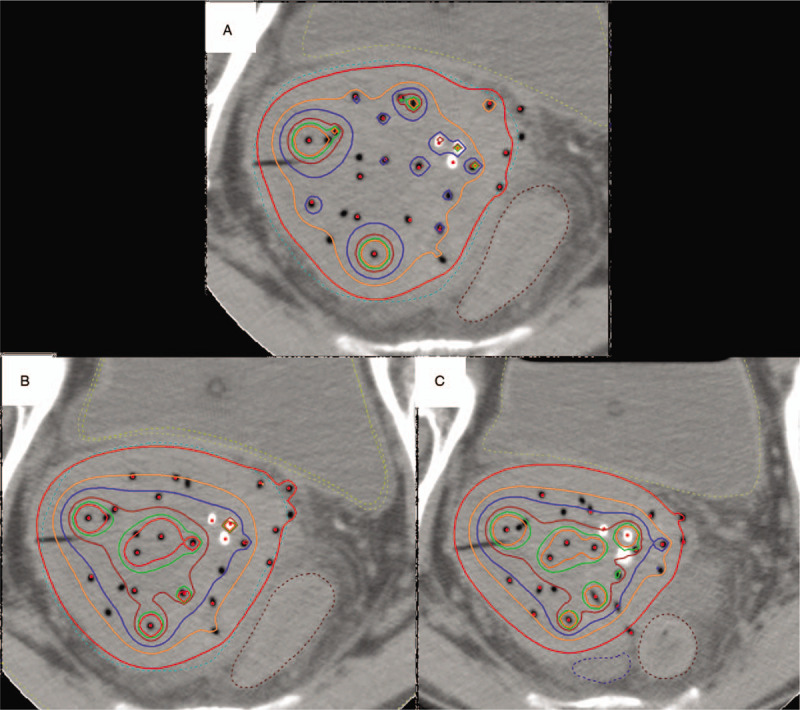
The dose distributions of 3 treatment plans. The treatment plans with isodose line of 100/150/200/300/400/500% of the prescribed dose red/beige/blue/brown/green/orange. (A) First-time ISBT, (B) second-time ISBT, (C) third-time ISBT. ISBT = high-dose-rate interstitial brachytherapy.

The needles were kept inserted until the third-time ISBT was finished and fixed with silicon putty for laboratory purposes (Fig. 1). Simulation computed tomography was performed at each ISBT to validate that the needled did not move from the first-time simulation computed tomography. Additionally, magnetic resonance imaging was performed after the first-time ISBT to evaluate the dose to OARs and HRCTV more accurately. In the second and third HDR-ISBT, the IIHD ISBT was performed, and the dose–volume histograms of all the 3 ISBTs are shown in Table 1. The prescribed reference dose per fraction (100% isodose) was 6 Gy in all ISBTs. Doses to the OARs (rectum/sigmoid/bladder, D0.1cc/0.2cc) and HRCTV D90/V100 were not significantly different between the first and the second/third ISBT, but HRCTV V200/300/400/500 was significantly higher in the second and third ISBT than in the first one (Table 1, Fig. 2). No acute adverse events caused by needle insertion and IIHD ISBT were detected. Additionally, complete response (Response Evaluation Criteria in Solid Tumors (RECIST) guideline version 1.1) was achieved, and no recurrence findings or late adverse events caused by HDR-ISBT have been detected for 2 years and 9 months.

**Table 1:**
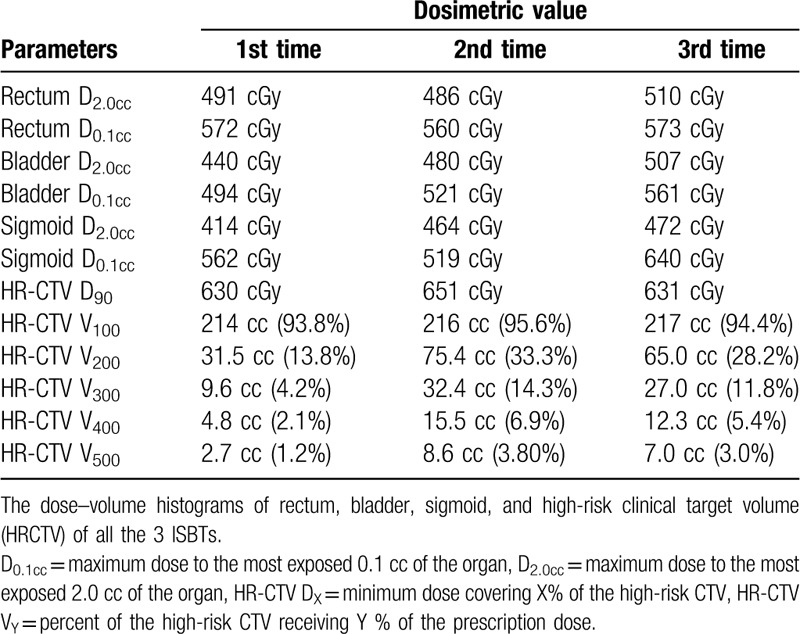
The dose–volume histograms of all the 3 ISBTs.

## 3 Discussion and Conclusions

A 54-year-old female with cervical cancer who received “IIHD policy” HDR-ISBT after EBRT of 50 Gy in 25 fractions was reported in this paper. To our knowledge, this is the first report that describes the effectiveness and safety of “IIHD policy” HDR-ISBT. Crane and Koay^[10]^ proposed the ablative dose painting SIP irradiation for large liver tumors. This strategy is reasonable for escalating the dose to the tumor, while meeting OAR dose limitations. However, respiratory movement has to be considered in abdominal external beam IIHD SIP irradiation, because high-dose irradiation to OARs is a significantly dangerous treatment. On the other hand, though there is a problem of internal organ motion,^[15]^ “IIHD policy” HDR-ISBT is a safer approach as far as peripheral dose is maintained around 6 Gy because needles for ISBT are inserted into the tumor.

Kuroda et al^[16]^ reported that 5-year overall survival rate of International Federation of Gynecology and Obstetrics stage IIIB (FIGO) cervical cancer patients treated by definitive radiation therapy was 52.4%. However, in stage III patients, bilateral parametrial infiltration and bulky disease (> 5 cm) had significantly higher recurrence rate.^[17]^ In this case, the transverse maximum diameter of the primary tumor was 10.5 cm with bilateral parametrial infiltration, and in recent study,^[18]^ disease-free survival rate of the patients who have stage IIIB cervical cancer more than 8 cm was 16.7%. Another previous study revealed that hypoxia may contribute to lower local control of bulky tumor. Hockel et al^[19]^ reported that hypoxic cervical cancer led to poorer outcome in RT, and large-sized tumor was related to hypoxia. In an animal experimental study by Epel et al^[20]^ it was demonstrated that boost irradiation to hypoxic area inside of the tumor significantly enhanced the local control. “IIHD policy” HDR-ISBT is a reasonable policy also in this point, and therefore, it would be a new strategy for bulky tumor.

There are 2 limitations in this report. One is that complete response might be obtained with “HDD policy” HDR-ISBT. Nevertheless, after the first-time ISBT, the tumor was still bulky, and the size was more than 8 cm. According to Pötter et al,^[21]^ when the HRCTV volume is larger than 30 cm^3^, HRCTV D90 needs to receive higher than 85 Gy. Nevertheless, EBRT 50 Gy in 25 fractions and 3 times of ISBT (HRCTV D90 of the first-time ISBT was 630 cGy) resulted in approximately 76 Gy, much less than 85 Gy. Therefore, it would be very difficult for the tumor to be controlled with “HDD policy.” To validate the effectiveness of this technique, a larger controlled study would be needed. The other is that the follow-up period is about 3 years, and then recurrence and late side effects can occur after this. Longer follow-up will be needed to conclude that “IIHD policy” HDR-ISBT is sufficiently safe.

In summary, this is the first report of “IIHD policy” HDR-ISBT for bulky cervical cancer. This policy can be the solution for bulky cervical cancer. Long-term follow-up will be needed to evaluate late side effects.

## Acknowledgments

The authors thank Editage (www.editage.jp) for English-language editing.

## Author contributions

All authors conceived of the study, and participated in its design and coordination and helped to draft the manuscript. They read and approved the final manuscript.
